# Retinal ischemic perivascular changes deteriorate vascular density in Alzheimer's disease

**DOI:** 10.1002/alz70856_101850

**Published:** 2025-12-24

**Authors:** Yufei Chen, William Robert Kwapong

**Affiliations:** ^1^ Xuanwu Hospital, Capital Medical University, Beijing, Beijing, China

## Abstract

**Background:**

Noninvasive retinal imaging may detect retinal ischemic perivascular lesion, RIPL (a retinal ischemic sign) associated with Alzheimer's Disease (AD) and may represent the interactive effect of increased white matter burden and decreased Aß_42_ concentration. We investigated the association of cerebral small vessel disease (SVD) markers and CSF concentration metrics with RIPL in AD.

**Method:**

This cross‐sectional study was conducted at the Neurology Department of Xuanwu Hospital, Capital Medical University from July 2024 to January 2025. Individuals who were Aß positron emission tomography (PET) positive and met the diagnosis of AD were eligible for inclusion and underwent an evaluation and diagnosis were enrolled. Individuals were who were cognitively unimpaired (CU) and Aß PET negative were enrolled as controls. All participants underwent magnetic resonance imaging (MRI) and optical coherence tomography (OCT)/OCT angiography (OCTA).

**Result:**

A total of 163 eyes from 92 AD (mean age 66.76 ± 8.67 years; 36.96 % males) and 194 eyes from 97 cognitively unimpaired controls (mean age 65.64 ± 8.13 years; 36.08 % males) were analyzed. Also, we found that RIPL was associated with lower Aß42 concentration (*r* = ‐1.01 95% CI ‐1.74 to ‐0.27; *p* = 0.007), increased burden of MTA (0.45 95% CI 0.09 to 0.82; *p* =  0.014) and periventricular WMH (0.47 95% CI 0.09 to 0.84; *p* =  0.014). When the cross‐product term of Aß_42_ and SVD markers was included in the regression model with RIPL as the outcome, the interaction was significant between Aß42 and periventricular WMH (ß = 0.011, *p* =  0.038).

**Conclusion:**

Increased periventricular WMH and decreased Aß42 concentration are associated with RIPL and AD patients with both pathologies are likely to have increased ischemia. Given the observed findings, further research is needed into RIPL's clinical utility in AD.

